# Impulsivity and Emotional Dysregulation Predict Choice Behavior During a Mixed-Strategy Game in Adolescents With Borderline Personality Disorder

**DOI:** 10.3389/fnins.2021.667399

**Published:** 2022-02-14

**Authors:** Ashley C. Parr, Olivia G. Calancie, Brian C. Coe, Sarosh Khalid-Khan, Douglas P. Munoz

**Affiliations:** ^1^Centre for Neuroscience Studies, Queen’s University, Kingston, ON, Canada; ^2^Department of Psychiatry, University of Pittsburgh, Pittsburgh, PA, United States; ^3^Division of Child and Youth Mental Health, Kingston Health Sciences Centre, Kingston, ON, Canada; ^4^Department of Biomedical and Molecular Sciences, Queen’s University, Kingston, ON, Canada

**Keywords:** Borderline Personality Disorder, emotional dysregulation, impulsivity, neuroeconomics, mixed-strategy, adolescence

## Abstract

Impulsivity and emotional dysregulation are two core features of borderline personality disorder (BPD), and the neural mechanisms recruited during mixed-strategy interactions overlap with frontolimbic networks that have been implicated in BPD. We investigated strategic choice patterns during the classic two-player game, Matching Pennies, where the most efficient strategy is to choose each option randomly from trial-to-trial to avoid exploitation by one’s opponent. Twenty-seven female adolescents with BPD (mean age: 16 years) and twenty-seven age-matched female controls (mean age: 16 years) participated in an experiment that explored the relationship between strategic choice behavior and impulsivity in both groups and emotional dysregulation in BPD. Relative to controls, BPD participants showed marginally fewer reinforcement learning biases, particularly decreased lose-shift biases, increased variability in reaction times (coefficient of variation; CV), and a greater percentage of anticipatory decisions. A subset of BPD participants with high levels of impulsivity showed higher overall reward rates, and greater modulation of reaction times by outcome, particularly following loss trials, relative to control and BPD participants with lower levels of impulsivity. Additionally, BPD participants with higher levels of emotional dysregulation showed marginally increased reward rate and increased entropy in choice patterns. Together, our preliminary results suggest that impulsivity and emotional dysregulation may contribute to variability in mixed-strategy decision-making in female adolescents with BPD.

## Introduction

Borderline Personality Disorder (BPD) is characterized by maladaptive decision-making tendencies, such as unstable relationships, self-harm behaviors, and substance use (for review, see Soloff et al., 2000; Dougherty et al., 2004; Rosval et al., 2006; Sebastian et al., 2014). Dimensional approaches propose that the symptoms of BPD reflect instantiations of an underlying predisposition toward impulsivity (i.e., actions without forethought or deliberation; Paliwal et al., 2014), emotion dysregulation, and interpersonal dysfunction (Chapman et al., 2008; Hallquist and Pilkonis, 2012; Scott et al., 2014; Beeney et al., 2018; Hallquist et al., 2018; Allen and Hallquist, 2020), which are transdiagnostic processes that contribute to maladaptive behaviors across several personality pathologies (see Allen and Hallquist, 2020 for a review). Personality and related psychiatric disorders, including BPD, often emerge and intensify during the adolescent period (Johnson et al., 2000a,b; Larsen and Luna, 2018). However, due in part to extant controversy surrounding diagnoses of personality disorders in adolescence (Volker et al., 2008; Coffey et al., 2011; Chanen and McCutcheon, 2013; Barker et al., 2015; Berenson et al., 2016; Krause-Utz et al., 2016; Maraz et al., 2016, p. 201) and the continued reliance on categorical classifications in the Diagnostic and Statistical Manual of Mental Disorders (DSM-5; Trull and Durrett, 2005; Widiger and Simonsen, 2005; Clark, 2007; American Psychiatric Association, 2013; Allen and Hallquist, 2020), little is known about how BPD symptoms emerge within the context of brain maturation, hampering the ability to develop effective diagnostic, preventative, and early intervention strategies (Chanen et al., 2007; Kaess et al., 2014).

Adolescence is a unique period of enhanced plasticity, marked by heightened risk-taking behaviors and impulsive decisions that can be adaptive (Spear, 2000), but can undermine survival and have adverse long-term consequences (e.g., risky sexual behavior, substance use, see Shulman et al., 2016 for review). Additionally, emotion regulation skills and strategies become refined through adolescence (Ochsner et al., 2012; Guassi Moreira et al., 2019). Contemporary models (Steinberg, 2010; Luna and Wright, 2016) conceptualize adolescent behavior as a normative peak in reward-driven and affective behaviors, which are adaptive for specializing the neurobiological pathways required for adult-like levels of cognitive and affective regulation (Spear, 2000; Luciana et al., 2012; Ochsner et al., 2012; Luna et al., 2015; Larsen and Luna, 2018; Guassi Moreira et al., 2019). Specifically these models propose a relative predominance of affective/reward systems over cognitive control systems (Shulman et al., 2016), biasing adolescent decision-making toward rewarding stimuli. Thus adolescence represents a period in which normative peaks in impulsivity and affective processing *already* undermine decision-making and cognitive control (Larsen and Luna, 2018), and aberrant developmental trajectories may contribute to the emergence of major psychopathologies during this period (Paus et al., 2008; Larsen and Luna, 2018). An increased propensity for impulsivity and risk-taking behaviors in adolescents with BPD can lead to adverse health outcomes (i.e., suicide and substance use; Kaess et al., 2014), and individuals diagnosed with BPD early in development may have more severe disease burden and poorer prognosis (Chanen et al., 2007; Kaess et al., 2014). Despite these findings, the majority of experimental studies concerning decision-making in BPD have been conducted in adults [a recent meta-analysis reported mean age of 27–30 years (Jeung et al., 2016; but see also Tay et al., 2017)], thus we know very little regarding the developmental pathways to dysfunctional decision-making in BPD (Kaess et al., 2014). We therefore focus on an adolescent cohort of BPD patients, with the aim of exploring how individual differences in impulsivity (that may be beyond the normative increases observed during adolescence) confer risk for maladaptive decision-making tendences in BPD.

Studies in adults with BPD have pointed to altered valuation of expected outcomes (Paret et al., 2017) predominantly assessed using delay discounting tasks, which measure an individual’s tendency toward immediate, short-term gratification, in addition to devaluation of long-term (delayed) rewards (Volker et al., 2008; Lawrence et al., 2010; Coffey et al., 2011; Barker et al., 2015; Berenson et al., 2016; Krause-Utz et al., 2016; Maraz et al., 2016; Lempert et al., 2019), reversal learning tasks (Berlin et al., 2005; Paret et al., 2016), and the Iowa Gambling Task (IGT; Haaland and Landro, 2007; Minzenberg et al., 2008; Black et al., 2009; Schuermann et al., 2011; LeGris et al., 2012; Cackowski et al., 2014), although findings on the latter are mixed (McCloskey et al., 2009; Gorlyn et al., 2013; Paret et al., 2017). Increased trait impulsivity (Skodol et al., 2002; Bornovalova et al., 2005; Lawrence et al., 2010; Barker et al., 2015) has been shown to contribute to increased discounting rates in BPD (Volker et al., 2008; Lawrence et al., 2010; Coffey et al., 2011; Krause-Utz et al., 2016), and variability in decision-making in healthy individuals (Franken et al., 2008; Penolazzi et al., 2012; Raio et al., 2020), including risk-taking behavior in adolescents (Romer et al., 2009). Increased levels of emotional dysregulation in BPD (e.g., assessed using the difficulties in emotion regulation scale DERS; Gratz and Roemer, 2004), particularly with regard to lacking access to emotion regulation strategies and impulse control difficulties (Ibraheim et al., 2017), may lead to exacerbated impulsivity, particularly in the context of negative affect (Koenigsberg et al., 2001; Chapman et al., 2008; Peters et al., 2013; Sebastian et al., 2013; Scott et al., 2014; Krause-Utz et al., 2016; Turner et al., 2017; Hallquist et al., 2018). Although developmentally appropriate assessment tools (including self-report, parent report, interview, and clinician report) have recently been validated to assess dysfunction in childhood and adolescence (Sharp et al., 2012a; Fonagy et al., 2015; Sharp and Fonagy, 2015), few experimental tasks that are sensitive to the pathological processes of BPD have been optimized for developmental populations.

Most tasks that have been used to probe decision-making in BPD have focused on processes involved during gambles with fixed outcomes and probabilities (i.e., gaining $1 immediately or $5 after some variable delay). Though these studies (as detailed above) have done much to advance our understanding of core decision-making deficits in BPD, and how BPD patients discount rewards according to their delays, these decisions are typically made in settings in which choices are independent of prior choices of the decision-maker and have consequences only for the decision-maker. In reality, decisions are rarely made in isolation and often made in highly complex social environments, which require a complex array of behaviors, the use of strategic tactics, and inference of the intentions of others (Lee, 2008), introducing a level of uncertainty in terms of value estimation. Neuroeconomic games that probe decision-making in ecologically valid (often interpersonal) contexts (Montague, 2007; Kishida et al., 2010; Hasler, 2012; King-Casas and Chiu, 2012; Sharp, 2012; Sharp et al., 2012a,b; Jeung et al., 2016; Robson et al., 2020) have revealed deficits in cooperative and trust behaviors in BPD, specifically related to perception of social norms and risk as compared to non-social risk (e.g., decisions involving fixed gambles; King-casas et al., 2008; Seres et al., 2009; Unoka et al., 2009; Franzen et al., 2011; Sharp, 2012; Preuss et al., 2016; Henco et al., 2020). Although these studies suggest changes in mentalization and cooperative decision-making processes in BPD, behavior during competitive interactions has not been investigated. Here, we explore how decisions made in a dynamic strategic context, which demands ongoing predictions of opponent’s choice behavior, differ in adolescents with BPD and as a function of impulsivity and emotional dysregulation.

During mixed-strategy games, such as rock-paper-scissors, each player’s actions and their associated outcomes change dynamically based on their opponent’s actions (von Neumann and Morgenstern, 1944; Seo and Lee, 2008; Vickery et al., 2011; Parr et al., 2020). On the one hand, it may be advantageous to adopt a mixed-strategy by choosing each of the three actions with equal frequency, but unpredictably from trial-to-trial. If both players do so, they approach the Nash equilibrium, and there is no incentive to deviate from this strategy unilaterally as departures could be exploited by their opponent (Nash, 1950; Fudenberg and Tirole, 1991). On the other hand, if one’s opponent deviates from the Nash equilibrium (e.g., by displaying preferences for one action or the other and/or serial dependence in choice patterns), one can benefit by exploiting these predictabilities, engaging mentalization processes involved in inferring the actions and mental states of others (Hampton et al., 2008; Vickery et al., 2015). Generally, individuals approach the Nash equilibrium, however, systematic deviations consistently emerge in normative studies of strategic decision-making in humans (Mikulić and Dorris, 2008; Vickery et al., 2011; Parr et al., 2020) and non-human primates (Barraclough et al., 2004; Lee et al., 2004; Thevarajah et al., 2009). Specifically, the win-stay/lose-shift (WSLS) bias emerges, consistent with the use of reinforcement learning (RL) processes (Cohen and Ranganath, 2007; Hampton et al., 2008), in which individuals are more likely to repeat previously successful actions (rewarded) and switch away from previously unsuccessful (unrewarded or punished) actions (Barraclough et al., 2004; Thevarajah et al., 2009; Vickery et al., 2011). Theoretically, these RL biases would be exploited by one’s opponent and result in decreased net gains, thus inhibition of RL biases is optimal, placing this task at odds with typical decision-making paradigms in which RL processes are adaptive for successful performance (i.e., the Iowa Gambling Task (Worthy et al., 2013); and multi-armed bandit tasks (Stojic et al., 2015). Mixed-strategy games engage limbic and affective systems in the brain (Sanfey et al., 2003; Dorris and Glimcher, 2004; Paulus et al., 2005; Seo and Lee, 2007; Seo et al., 2009; Vickery and Jiang, 2009; Abe and Lee, 2011; Vickery et al., 2011; Harlé and Sanfey, 2012; Katahira et al., 2015; Parr et al., 2020), and involve frontolimbic networks that overlap with those implicated in BPD (Lyoo et al., 1998; Tebartz Van Elst et al., 2003; Silbersweig et al., 2007; P. Soloff et al., 2008; Ruocco et al., 2013; Schulze et al., 2016). Importantly, this paradigm is sensitive to several domains of dysfunction in BPD. First, Impulsivity, given its role in decision-making under uncertainty (Dickman, 1993; Zermatten et al., 2005; Paliwal et al., 2014; Chase et al., 2017; Sharma et al., 2017), may play a role in learning and updating the value of available actions (action-outcome contingencies) that change dynamically based on reinforcement history and opponent’s behaviors (Lee and Seo, 2007; Vickery et al., 2011). Second, emotional dysregulation, which has been shown to exacerbate impulsivity in BPD (Chapman et al., 2008; Sebastian et al., 2013; Neville et al., 2021), may play a role in integrating affective sources of information to update value representations (Paulus et al., 2005). Here, we characterize how individual differences in impulsivity and emotional dysregulation affect choice behavior during a mixed-strategy game in adolescents with BPD.

Given the novelty of this paradigm and the general lack of experimental findings in adolescent mixed-strategy decision-making and in adolescent BPD, this study was largely exploratory in nature. As a first-of-its kind study, we tested the possibility that BPD pathology could lead to either a detriment or a benefit to task performance. Pathological processes in BPD (namely, impulsivity and emotional dysregulation) could affect strategic choice behavior in several ways. Impaired affective and RL processes could contribute to strategic choice behavior through outcome evaluation processes including; (1) Blunted reward prediction error (RPE) and/or affective signaling (Hüpen et al., 2020; Neville et al., 2021) that could lead to insensitivity to changing reward contingencies and/or a deficit updating the value of actions on a trial-by-trial basis, which in this context, may actually result in *fewer* choice biases (and therefore, potentially enhanced performance as one may better evade exploitation by the opponent); and/or (2) Exacerbated RPE and/or affective signals could lead to *amplified* biases (and therefore, potentially diminished performance as the opponent would exploit these biases as they emerge), either of which may be more prominent for either positive (WS) and/or negative (LS) outcomes (Schuermann et al., 2011; Paret et al., 2016; Neville et al., 2021). We hypothesized that group differences would be more pronounced in individuals with higher levels of impulsivity in either group, measured using the BIS (Patton et al., 1995), and emotion dysregulation in the BPD group, measured using the DERS (Gratz and Roemer, 2004). Impulsivity (Stanford et al., 2009) and emotional dysregulation (Gratz and Roemer, 2004) are multifaceted constructs, and differential patterns of associations among impulsivity and DERS subscales have been associated with distinct clinically relevant outcomes (Chapman et al., 2008; Neumann et al., 2010), including externalizing and internalizing characteristics in adolescents (Neumann et al., 2010). Because our study is novel and exploratory in nature, we first interrogated relationships at the level of total BIS and DERS scores, and then examine the contribution of each subscale to strategic choice behavior. Last, to gain insight into whether any observed changes are related to deliberative strategies (as opposed to non-strategic randomness or noise, for example) we further conducted exploratory analyses to assess whether individual differences in self-report strategies contribute to the observed group differences in choice behavior and relationships between impulsivity (both groups) and difficulties in emotional regulation (BPD group).

## Materials and Methods

### Participants

Female adolescents with BPD and age- and sex- matched control adolescents participated in an experiment that examined choice patterns during the mixed-strategy game, Matching Pennies (a two-choice variant of Rock-Paper-Scissors). Twenty-eight female adolescents (mean age: 16.3 years, ±1.3, range: 14–18) who met criteria for BPD (assessed by the Structured Clinical Interview for DSM-5 Personality Disorders (SCID-PD; Pfohl et al., 1997) were recruited from the Dialectical Behavioral Therapy (DBT) group at Kingston Health Sciences Center outpatient mood and anxiety clinic at Hotel Dieu Hospital by co-author SKK. One BPD participant was excluded due to poor performance that indicated a lack of understanding of the task procedures (various performance measures including overall reward rate falling ≥3 SD from the group mean). Final analyses included twenty-seven female participants with BPD (mean age: 16.4 years, ±1.3, range: 14–18) and twenty-seven female control participants (mean age: 15.8 years, ±1.6, range: 14–18) recruited from the community in Kingston, ON, Canada. This study was approved by the Queen’s University Human Research Ethics Board and was in accordance with the *Canadian Tri-Council Policy Statement on Ethical Conduct for Research Involving Humans* and the principles of the *Declaration of Helsinki*. All participants gave informed consent and were compensated for their time. Both BPD and control participants completed an evaluation of impulsivity [Barratt Impulsiveness Scale (BIS-11; Patton et al., 1995)] as well as a post-game questionnaire (developed for the current study, see section “Materials and Methods”) assessing how participants approached the strategic game (referred to as section “Strategic Assessment”). Mean scores are shown in Table 1. BPD patients underwent an evaluation of borderline-typical symptomology [Borderline Symptom List (BSL-23); Bohus et al., 2007] and emotional dysregulation [Difficulties in Emotional Regulation Scale (DERS); Gratz and Roemer, 2004]. Mean clinical scores for the BPD group are shown in Table 2. All BPD participants remained on their regular medication regimes throughout the duration of the study (medication information is shown in Table 2). Groups did not differ in terms of age (*BPD Mean* = 16.4 years, *SD* = 1.28, *CTRL Mean* = 15.8 years, *SD* = 1.55, *t* = –1.44, *p* = 0.16), or handedness (*BPD*: 24 righthanded; *CTRL*: 26 righthanded; *t* = –0.85, *p* = 0.40, *p* = 0.39).

**TABLE 1:** Descriptive statistics of participants.

	BPD	*F* Control			
	Mean (SE)	Mean (SE)	β	*T*	*p*
**Sample size**	27	27			
**Age**					
Mean	16.37 (0.24)	15.81 (0.29)	–0.39	–1.44	0.16
Range	14–18	14–18			
**Handedness**	24 R (3 L)	26 R (1 L)	–0.11	–0.85	0.40
**BIS scores**					
Total	80.81 (1.88)	64.55 (2.15)	**–1.28**	**–5.87**	**<0.001**
Motor	19.29 (0.65)	15.44 (0.62)	**–1.06**	**–4.39**	**<0.001**
Cognitive instability	8.88 (0.31)	6.48 (0.37)	**–1.07**	**–4.61**	**<0.001**
Attention	14.29 (0.52)	9.77 (0.54)	**–1.26**	**–5.79**	**<0.001**
Self-control	15.11 (0.65)	12.29 (0.64)	**–0.88**	**–3.51**	**<0.001**
Cognitive complexity	13.62 (0.43)	12.40 (0.48)	**–0.55**	**–2.04**	**0.05**
Perseverance	9.59 (0.39)	7.77 (0.52)	**–0.74**	**–2.81**	**0.01**
**Strategic assessment**					
Attention to outcome	3.55 (0.23)	4.30 (0.13)	**0.77**	**2.95**	**0.005**
Random strategy	2.63 (0.23)	2.63 (0.17)	–0.50	–1.84	0.07
Predictive strategy	3.92 (0.24)	4.00 (0.18)	0.11	0.38	0.70
Human vs. computer	4.03 (0.22)	3.78 (0.21)	–0.19	–0.66	0.51
Spatial-based strategy	3.48 (0.17)	3.41 (0.17)	0.04	0.13	0.89
Color-based strategy	3.41 (0.19)	3.67 (0.17)	0.26	0.92	0.36

*Data are reported as means (SE) unless otherwise indicated. BPD, borderline personality disorder; F, female; BIS, Barratt Impulsiveness Scale (BIS-11). P values are Bonferroni adjusted. Independent samples t-test. β coefficients are standardized.*

*Significant test results are highlighted in bold.*

**TABLE 2:** Clinical scores and medication information for BPD participants.

	BPD
	**Mean (SE)**
BSL^a^	53.52 (4.88)
DERS^b^	
Total	119.26 (3.74)
Non-acceptance	22.46 (1.10)
Goals	18.65 (0.66)
Impulse control	20.69 (0.96)
Awareness	16.23 (0.90)
Strategies	27.38 (1.18)
Clarity	13.84 (0.48)

**Medication (Class)*^c^***	**N (% of participants)**

Stimulant	6 (24)
Non-Stimulant	3 (12)

*Questionnaire data are reported as means (SE), while ADHD medication data are reported as number (percentage) of participants on that class of medication. BSL, Borderline Symptom List; DERS, Difficulties in Emotional Regulation Scale. Stimulant refers to Concerta, Methlyphenidate, Ritalin, or Biphentin. Non-Stimulant refers to Intuniv or Straterra. Note that percentage of participants is based on the number of participants we have medication data for (N = 25).*

*^a^Data not available for two patients (N = 25).*

*^b^Data not available for one patient (N = 26).*

*^c^Data not available for two patients (N = 25).*

### Task Procedures

#### Strategic Decision-Making Task

**FIGURE 1:**
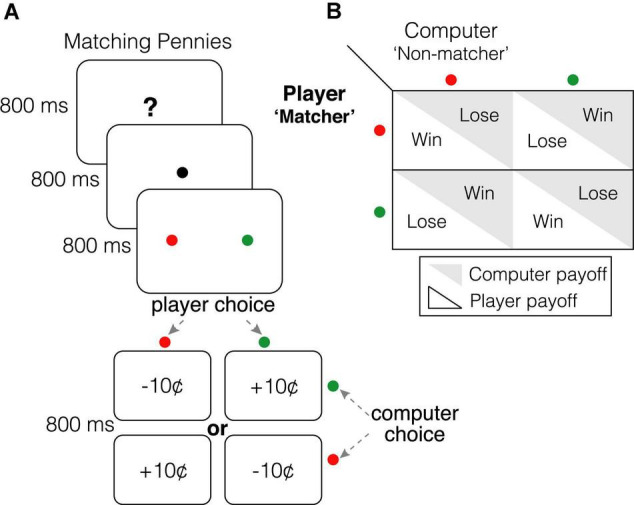
Matching Pennies. **(A)** Participants were informed that they were competing in Matching Pennies against a dynamic computer opponent that analyzed their behavior and exploited predictabilities in their response patterns (see algorithm 2 in Lee et al., 2004 and Parr et al., 2020). Participants played the role of the “matcher” while the computer opponent played the “non-matcher”; if both players chose the same colored target, participants received a “10¢” reward for that trial. Otherwise, participants lost “10¢” for that trial. They were instructed to win as much money as possible. **(B)** Payoff matrix for each player.

Participants competed in a color-based version of Matching Pennies against a dynamic computer opponent that exploited predictabilities in player choice patterns (Figure 1A). Participants played the role of the *matcher*, while the computer opponent played the role of the *non-matcher—*if both players chose the same colored target, the participant won $0.10; otherwise, the participant lost $0.10 (Figure 1B; players were endowed with 30 credits at the beginning of the session). Briefly, the competitive algorithm employed by our computer opponent was based on algorithm 2 in Barraclough et al. (2004), and performed a statistical analysis of participants’ historical sequence of choices (including both leftward/rightward target and red/green target) and associated payoffs (rewarded or unrewarded) to uncover systematic biases (Barraclough et al., 2004; Parr et al., 2020). Participants were informed of the rules of the game, were aware that they were playing a strategic game against a dynamic, competitive, computer opponent, and were instructed to win as much money as possible. If participants approached the Nash equilibrium (e.g., by successfully evading exploitation by the opponent and/or randomizing over choice patterns), they would win approximately 50% of trials.

Participants were informed that monetary compensation for the current study was dependent on task performance (cumulative amount of reward earned) during Matching Pennies. However, independent of task performance, each participant was compensated with a $30 gift card.

#### Experimental Design and Timing

Participants completed four runs (each consisting of 150 trials for a total of 600 trials) of Matching Pennies (Figure 1A). Each trial was 3200 ms in duration and started with 800 ms of a task identification period followed by 800 ms of a fixation period. Next, two visual targets, one green and one red, appeared for 800 ms at an eccentricity of 6.5° to the left and the right of the fixation point, during which participants indicated their choice of target with a saccadic eye movement. Finally, the outcome of each trial (monetary reward) was revealed during an 800 ms period. Participants were presented with a 20 s long fixation period at the beginning and end of each run. Each run was 8 min and 40 s in duration, and the total session time was approximately 60 min (including breaks between runs and approximately 15 min for questionnaires). Each participant completed 20 practice trials at the beginning of the session.

Importantly, the location of the colored targets was pseudo-randomized, appearing on each side of the screen 50% of the time. Participants were instructed to maintain fixation prior to the appearance of the targets. Saccades made prior to the appearance of the targets were considered anticipatory trials and those made >800 ms following the appearance of the targets were considered non-response trials.

#### Recording and Apparatus

Monocular eye position data was recorded at 500 Hz using the EyeLink 1000 Version 5.1 table mounted eye-tracking device (SR-Research Ltd., Mississauga, ON, Canada). The monitor, infrared illuminator, and camera were positioned 60 cm away from central gaze, and the right eye was recorded. Participants were situated in a mounted chin rest, stabilizing the head and limiting motion during each trial. All visual stimuli were presented and behavioral responses acquired using custom MATLAB v7.9 programs (The Mathworks Inc., Natick, MA, United States) and Psychophysics Toolbox v3 (Brainard, 1996; Pelli, 1996) running on a PC. Visual stimuli were presented on an adjustable 17-inch LCD monitor at a screen resolution of 1280 × 1024 pixels that had a refresh rate of 60 Hz. At the beginning of each run, eye position was calibrated using a five-point calibration routine set to within 1° of the visual target. Participants indicated their choices by making a saccadic eye movement that corresponded to the location of the desired visual target. Saccades were recorded if eye movement amplitude exceeded a 7.5° fixation window. Eye tracking was used as it is very accurate and eye movements do not include any biomechanical lags that can impact limb motion.

#### Performance Variables

The following behavioral variables were examined: (1) the probability of reward [p(rew)], which served as a proxy for overall task performance.

#### Analysis of Win-Stay, Lose-Shift Tendencies

We also measured the extent to which participants’ choices depended on a history of previous choices and outcomes by calculating (2) the probability of using the win-stay-lose-shift strategy [p(wsls)]. Win-stay [p(ws)] refers to the probability of selecting the same target as the previous trial if it was rewarded, and lose-shift [p(ls)] refers to the probability of switching to the opposite target following a previously unrewarded trial (Barraclough et al., 2004; Lee et al., 2004; Parr et al., 2020). Choices for each participants following reward or loss for each trial, *t*, were analyzed as follows:


$$win\left(t\right)=\left\{ \begin{array}{cc} 0 & foranon-responsetrialorlosstrial\\ 1 & forarewardedtrial \end{array}\right.$$

$$loss\left(t\right)=\left\{ \begin{array}{cc} 0 & foranon-responsetrialorrewardedtrial\\ 1 & foranon-rewardedtrial \end{array}\right.$$

Following rewarded trials [win(*t*) = 1], a win-stay event was counted for trial *t* + 1 if participants chose the same target as the previous trial (trial *t*).


$$stay\left(t+1\right)=\left\{ \begin{array}{c} 0foranonresponsetrialorforswitchingtothe\\ \;oppositetarget\\ 1forchoosingthesametargetfollowingreward \end{array}\right.$$

Following loss trials [loss(*t*) = 1], a lose-shift event was counted for trial *t* + 1 if participants chose the opposite target as the previous trial (trial *t*).


$$shift\left(t+1\right)=\left\{ \begin{array}{c} 0foranonresponsetrialorforchoosingthe\\ \;sametarget\\ 1forchoosingtheoppositetargetfollowingloss \end{array}\right.$$

Win-stay was calculated as:


$$P\left(stay|win\right)=\frac{\sum stay}{\sum win}$$

Lose-shift was calculated as:


$$P\left(shift|loss\right)=\frac{\sum shift}{\sum loss}$$

Win-stay, lose-shift was calculated as:


$$P\left(WSLS\right)=\frac{\sum P\left(stay|win\right)+P\left(shift|loss\right)}{\sum win+loss}$$

#### Entropy Analyses

To quantify the degree of randomness in participants’ choice patterns, we calculated (3) entropy using participants choice sequences (independent of trial outcome, win or loss), termed choice entropy (Cover and Thomas, 1991). We also calculated (4) entropy using the choice sequence of the two players (which is equivalent to using trial outcome), termed choice-outcome entropy (see Lee et al., 2004 for details).


$$H=-\sum_{i=1}^{k}p_{i}\log_{2}p_{i}\left(\text{bits}\right)$$

Calculating the entropy based on the participant’s choice (choice entropy) in three successive trials, there are a total number of eight possible outcomes (*k* = 2^3^= 8), and the maximum entropy is 3 bits. When entropy is calculated based on the participant’s choice sequence in three successive trials, as well as the computer opponent’s choice sequence in two prior trials (choice-outcome entropy), there are a total number of 32 possible outcomes (*k* = 2^5^ = 32), and the maximum entropy is 5 bits. Entropy was log transformed for all analyses.

Both p(wsls) and entropy variables were computed separately for both color and spatial domains (i.e., if participants chose the righthand target following a rewarded outcome on the right, this would be considered spatial wsls bias).

#### Response Time Variables

We additionally recorded saccadic reaction times (SRT), measured from stimulus appearance to onset of the first saccade away from fixation (outside of the 7.5 degree window), and calculated median SRT (overall), as well as median SRT following rewarded trials and loss trials (separately). Additionally, we calculated the coefficient of variation in SRT (CV; *CV* = SD/Mean * 100), the percentage of anticipatory trials, and the percentage of non-response trials. Anticipatory and non-response trials were censored prior to calculating all other behavioral variables above. Additionally, trials with SRTs that exceeded 3 SD from the mean were excluded prior to calculating median SRT and CV for all analyses. Note that two participants were removed from CV analyses due to values that exceeded 3 SD from the mean (one BPD participant and one CTRL participant leaving 26 participants in each group included in all CV analyses below).

### Assessments

#### Barratt Impulsiveness Scale

In both groups, we investigated whether individual variation in impulsivity corresponded to strategic choice behaviors. The Barratt Impulsiveness Scale (BIS-11; Mean scores for each subscale are shown in Table 1) is a well validated tool (Barratt, 1959; Patton et al., 1995; Stanford et al., 2009), and six first-order factors (sub-traits) have been identified (Patton et al., 1995) that reflect impulsivity across the following domains: (1) Attention (e.g., “I concentrate easy,” “I am a steady thinker,” “I am restless in lectures”); (2) Motor (e.g., “I do things without thinking,” “I act on impulse/on the spur of the moment,” “I buy things on impulse”); (3) Self-control (e.g., “I plan tasks carefully,” “I am self-controlled,” “I say things without thinking”); (4) Cognitive complexity (e.g., “I like to think about complex problems,” “I am more interested in the present than the future,” “I like puzzles”); (5) Perseverance (e.g., “I can only think about one thing at a time,” “I am future oriented,” “I change jobs”); and (6) Cognitive instability (e.g., “I have racing thoughts,” “I change hobbies,” “I often have extraneous thoughts when thinking”; Patton et al., 1995; Stanford et al., 2009). Higher scores on each scale indicate greater impulsivity (mean scores for each subscale are shown in Table 1).

#### Difficulties in Emotional Regulation

In the BPD group, we investigated whether choice patterns changed as a function of emotional dysregulation. The Difficulties in Emotional Regulation Scale (DERS; Gratz and Roemer, 2004; mean scores for each subscale are shown in Table 2) measures functioning across several domains: (1) Non-acceptance of emotional responses (non-acceptance; e.g., “When I’m upset, I become irritated with myself for feeling that way”); (2) Difficulties engaging in goal-directed behavior (goals; e.g., “When I’m upset, I have difficulty thinking about anything else”); (3) Impulse control difficulties (impulse control; e.g., “When I’m upset, I have difficulty controlling my behaviors”); (4) Lack of emotional awareness [awareness; e.g., “When I’m upset, I (do not) acknowledge my emotions”]; (5). Limited access to emotion regulation strategies (strategies; e.g., “When I’m upset, I start to feel very bad about myself”); and (6) Lack of emotional clarity (clarity; e.g., “I have difficulty making sense out of my feelings”; Gratz and Roemer, 2004). Higher scores indicate greater emotional dysregulation (mean scores for each subscale are shown in Table 2). Importantly, DERS data were not collected in control participants, thus we do not present group comparisons for this measure.

#### Strategic Assessment

In both groups, we administered a post-game questionnaire that was developed for the current study to assess whether the way in which participants approached the mixed-strategy game affected strategic choice patterns. The questionnaire was comprised of the following items: (1) Attention to outcome: “I paid attention to whether I was rewarded or not, and changed by strategy when I wasn’t being rewarded (or when I was)”; (2) Spatial strategy: “I decided which target to choose before the targets appeared on the screen (I chose based on left or right side of the screen)”; (3) Color strategy: “I decided which target to choose based on the color of the targets”; (4) Random strategy: “I found that it was easy to choose randomly”; (5) Exploit opponent: “I tried to predict what my opponent would do”; and (6) Human versus computer: “I think I would play differently if I was playing against a human opponent as opposed to a computer.” Each item was rated on a 5-point scale with 1 representing “Strongly Disagree” and 5 representing “Strongly Agree,” and we assessed the relationship between each item and choice behavior (mean scores for each item are shown in Table 1).

### Data Analysis

Statistical analyses were carried out with custom MATLAB programs version 9.3 (The MathWorks Inc., Natick, MA, United States) and R version 3.5.2 *via* RStudio version 1.2.1 (R Development Core Team, 2007; RStudio Team, 2020). All continuous variables were z-scored prior to statistical analyses and z-scores are shown in scatterplots to allow for visualization across different assessments.

#### Group Differences in Task Performance

We first evaluated whether there were significant differences between groups on the dependent variables affecting Matching Pennies performance, including (1) reward rate; (2) choice biases; (3) win-stay, lose-shift biases; (4) choice entropy; and (5) choice-outcome entropy. The latter four measures were calculated for both the spatial and color domains. Performance data were considered significant at *p* < 0.01 to account for multiple comparisons (Bonferroni correction: 0.05/5 performance variables, corrected alpha = 0.01).

Next, we evaluated differences between groups on the dependent variables affecting response times, including (1) median SRT (overall); (2) SRT following rewarded and unrewarded outcomes; (3) CV; and (4) % anticipatory trials. Data were considered significant at *p* < 0.01 to account for multiple comparisons (Bonferroni correction: 0.05/4 behavioral variables, corrected alpha = 0.01).

To explore whether any group differences were being driven by BPD participants who were taking stimulant medication [to treat symptoms of attention deficit hyperactivity disorder (ADHD)], we further tested for differences among BPD participants with a diagnosis of ADHD (*n* = 14) and those prescribed stimulants for comorbid ADHD (*n* = 6; see Table 2).

In all cases, linear regression models (LM; *stats* package in R) were conducted to investigate between-group differences on individual dependent variables. Because this was a developmental sample, age was included as a covariate in all models. All continuous variables were z-scored prior to analyses, and standardized beta coefficients are reported.

#### Associations With Barratt Impulsiveness Scale and Difficulties in Emotional Regulation Scores

We next conducted linear regression models to examine the relationship between impulsivity (each subscale of the BIS) and choice behaviors, and whether this differed as a function of group (BPD versus control). We first tested for assessment by group interactions, and interaction terms were removed from the final models when not significant. In the BPD group, we examined the relationship between emotional dysregulation (DERS) and choice behaviors (as DERS was not conducted in control participants). In all cases, age was modeled as a covariate.

#### Associations With Strategic Assessment (Exploratory Analyses)

We conducted linear regression models to explore the relationship between self-reported strategies (Strategic Assessment, see section “Materials and Methods”) and choice behaviors, and whether this differed as a function of group (BPD versus control). We tested for interactions with group in the same fashion as above and age was modeled as a covariate.

To explore whether any associations were being driven by BPD participants who were taking stimulant medication (*n* = 6) to treat symptoms of attention deficit hyperactivity disorder (ADHD, *n* = 6), we repeated the models in the BPD group testing for assessment (BIS, Strategic Assessment and DERS) by stimulant/ADHD diagnosis interactions, and interaction terms were removed from the final models when there were no significant interaction terms and effects are reported modeling each as a covariate.

Correlation matrices (ggcorrplot, R) were computed for visualization of correlations among variables and assessments in each group.

## Results

### Group Differences in Task Performance

#### Probability of Reward

**FIGURE 2:**
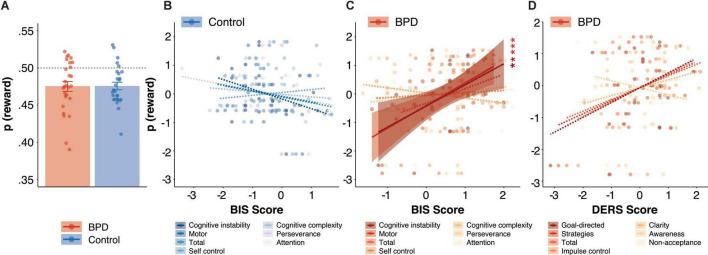
Reward rate during Matching Pennies. **(A)** Group differences in reward rate. The relationship between reward rate and BIS impulsivity scores in **(B)** control and **(C)** BPD participants. **(D)** The relationship between reward rate and DERS emotion dysregulation scores in BPD participants. In panels **(B–D)**, *z*-scores are shown to allow for visualization across different subscales, standardized beta coefficients from linear models are reported, and solid and dashed lines indicate significant and non-significant regressions, respectively, and reported *p* values are Bonferroni corrected. ** *p* < 0.01, *** *p* < 0.001.

Borderline personality disorder and control participants were comparable on overall probability of reward [p(rew); Figure 2A, Table 3, *p* > 0.05] which was significantly lower than 0.50 in both BPD (*M* = 0.47, *SE* = 0.006; *t* = –3.76, *p* < 0.001) and control participants (*M* = 0.47, *SE* = 0.005; *t* = –4.74, *p* < 0.001), and did not differ between groups (β = 0.05, *t* = 0.17, *p* = 0.86, *p_*Bonferroni*_* = 1.00).

**TABLE 3:** Matching Pennies task performance.

	BPD	*F* control				
Measure	Mean (SE)	Mean (SE)	β	*T*	*p value*	*p*_*Bonferroni*_
p (rew)	0.47 (0.006)	0.47 (0.005)	0.05	0.17	0.86	1.00
**Spatial biases**						
p (wsls)	0.55 (0.01)	0.56 (0.008)	0.12	0.41	0.68	1.00
p (ws)	0.51 (0.01)	0.52 (0.01)	0.23	0.83	0.41	1.00
p (ls)	0.60 (0.01)	0.60 (0.01)	–0.03	–0.10	0.92	1.00
p (right)	0.51 (0.009)	0.53 (0.007)	0.26	0.94	0.35	1.00
choice entropy	2.94 (0.01)	2.93 (0.01)	–0.09	–0.32	0.75	1.00
choice-outcome entropy	4.78 (0.02)	4.81 (0.01)	0.27	0.96	0.34	1.00
**Color Biases**						
p (wsls)	0.51 (0.005)	0.53 (0.007)	**0.64**	**2.42**	**0.02**	0.10
p (ws)	0.53 (0.007)	0.54 (0.01)	0.39	1.41	0.16	0.80
p (ls)	0.50 (0.006)	0.53 (0.009)	**0.62**	**2.31**	**0.03**	0.15
p (green)	0.51 (0.004)	0.51 (0.004)	–0.12	–0.44	0.66	1.00
choice entropy	2.99 (0.01)	2.99 (0.02)	–0.33	–1.18	0.24	1.00
choice-outcome entropy	4.95 (0.03)	4.93 (0.03)	–0.47	–1.72	0.09	0.45
**Response variables**						
Median RT (ms)	255 (5.43)	262 (5.13)	0.29	1.11	0.27	1.00
Median RT after reward	–1.94 (1.41)	–1.46 (1.41)	–0.13	–0.43	0.67	1.00
Median RT after loss	–3.66 (1.59)	–1.91 (1.12)	0.14	0.44	0.66	1.00
CV (%)	32.88 (1.34)	29.72 (1.23)	–0.50	–1.99	**0.05**	0.20
% anticipatory	6.71 (0.69)	3.32 (0.49)	**–0.92**	**–3.73**	**<0.001**	**<0.001**
% non-response	1.42 (0.36)	.46 (0.10)	**–0.67**	**–2.50**	**0.02**	**0.08**

*Data are reported as means (SE) unless otherwise indicated. BPD, borderline personality disorder; F, female; p (rew), probability of reward on average; p (right), probability of choosing the right hand target on average; p (green), probability of choosing the green target on average; P (WSLS), probability of win-stay, lose-shift strategy. Choice entropy, entropy in choice sequence in three successive trials and the choice in the next trial. Choice-outcome entropy, entropy in choice sequence of two players in two successive trials and the subject’s choice in the next trial. RT, reaction time; ms; milliseconds. Statistics reflect results from linear regression models, all β coefficients are standardized.*

*Significant test results are highlighted in bold.*

#### Overall Color Biases

The probability of choosing green [p(green), indicating color bias independent of reward history] was significantly higher than 0.50 in both BPD (*M* = 0.51, *SE* = 0.004; *t* = 3.91, *p* < 0.001) and control participants (*M* = 0.51, *SE* = 0.004; *t* = 3.41, *p* = 0.002), and did not differ between groups (β = –0.12, *t* = –0.44, *p* = 0.66, *p_*Bonferroni*_* = 1.00). Likewise, choice entropy (color) did not differ between groups (β = –0.33, *t* = –1.18, *p* = 0.24, *p_*Bonferroni*_* = 1.00).

#### Overall Spatial Biases

The probability of choosing right [p(right), indicating spatial bias independent of reward history] was significantly higher than 0.50 in control participants (*M* = 0.53, *SE* = 0.007; *t* = 4.16, *p* < 0.001) but not BPD participants (*M* = 0.51, *SE* = 0.009; *t* = 1.86, *p* = 0.07), although this was not significantly different between groups (β = 0.26, *t* = 0.94, *p* = 0.35, *p_*Bonferroni*_* = 1.00). Likewise, choice entropy (spatial) did not differ between groups (β = –0.09, *t* = –0.32, *p* = 0.75, *p_*Bonferroni*_* = 1.00).

#### Color-Based Win-Stay, Lose-Shift Bias

**FIGURE 3:**
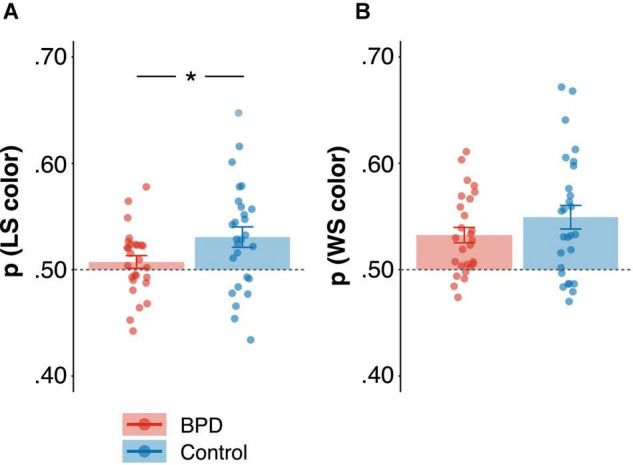
Group differences in the probability of win-stay, lose-shift [p(wsls)] in the color domain. **(A)** Probability of lose-shift [p(ls)] and **(B)** Probability of win-stay [p(ws)]. Standardized beta coefficients from linear models are reported, and reported *p* values are uncorrected. * *p* < 0.05.

The probability of win-stay, lose-shift in the color domain [p(WSLS color)] was significantly higher than 0.50 in both BPD (*M* = 0.52, *SE* = 0.005; *t* = 3.74, *p* < 0.001) and control participants (*M* = 0.54, *SE* = 0.007; *t* = 5.18, *p* < 0.001), however, p(WSLS color) was lower in the BPD group as compared to the control group (Table 3; β = 0.64, *t* = 2.42, *p* = 0.02, *p_*Bonferroni*_* = 0.10), though this failed to reach criteria for multiple comparisons despite the relatively large effect size. Nonetheless, follow-up analyses revealed that this was driven by a decrease in the probability of lose-shift [p(LS color)] in the BPD participants relative to controls (Figure 3A and Table 3; β = 0.62, *t* = 2.31, *p* = 0.03), and the BPD group did not differ significantly from 0.50 (*M* = 0.50, *SE* = 0.006; *t* = 1.18, *p* = 0.25) but control participants did (*M* = 0.53, *SE* = 0.01; *t* = 3.16, *p* = 0.004). The probability of win-stay [p(WS color)] on the other hand was significantly higher than 0.50 in both BPD (Figure 3B and Table 3; *M* = 0.53, *SE* = 0.007; *t* = 4.53, *p* < 0.001) and control participants (*M* = 0.55, *SE* = 0.01; *t* = 4.43, *p* < 0.001), and did not differ significantly between groups (Table 3; β = 0.39, *t* = 1.41, *p* = 0.16). Although there was a trend, choice-outcome entropy (color) did not differ between groups (β = –0.47, *t* = –1.72, *p* = 0.09, *p_*Bonferroni*_* = 0.45).

#### Spatial-Based Win-Stay, Lose-Shift Bias

The probability of win-stay, lose-shift in the spatial domain [p(WSLS spatial)] was significantly higher than 0.50 in both BPD (*M* = 0.56, *SE* = 0.01; *t* = 4.44, *p* < 0.001) and control participants (*M* = 0.57, *SE* = 0.009; *t* = 7.38, *p* < 0.001), and p(WSLS spatial) did not differ between groups (Table 3; β = 0.12, *t* = 0.41, *p* = 0.68, *p_*Bonferroni*_* = 1.00). Follow-up analyses revealed that the probability of lose-shift [p(LS) spatial] differed significantly from 0.50 in both BPD participants (*M* = 0.60, *SE* = 0.01; *t* = 5.57, *p* < 0.001) and control participants (*M* = 0.60, *SE* = 0.01; *t* = 5.84, *p* < 0.001) and p(LS spatial) did not differ between groups (Table 3; β = –0.03, *t* = –0.10, *p* = 0.92). The probability of win-stay [p(WS) spatial], on the other hand, did not differ from 0.50 in either BPD participants (*M* = 0.51, *SE* = 0.01; *t* = 0.80, *p* = 0.43) or control participants (*M* = 0.52, *SE* = 0.01; *t* = 1.61, *p* = 0.12), and did not differ between groups (Table 3; β = 0.23, *t* = 0.83, *p* = 0.41). Likewise, choice-outcome entropy (spatial) did not differ between groups (β = 0.27, *t* = 0.96, *p* = 0.34, *p_*Bonferroni*_* = 1.00).

No performance variables differed as a function of stimulant medication or ADHD diagnosis in BPD (all *p* > 0.05). We did not observe any significant effects of age (all *p* > 0.05), and age was modeled as a covariate in all group analyses. Finally, we did not observe any significant associations with overall BPD pathology as measured by the Borderline Symptoms List (BSL; Table 2, see Supplementary Section).

### Response Time Variables and Their Modulation by Outcome

**FIGURE 4:**
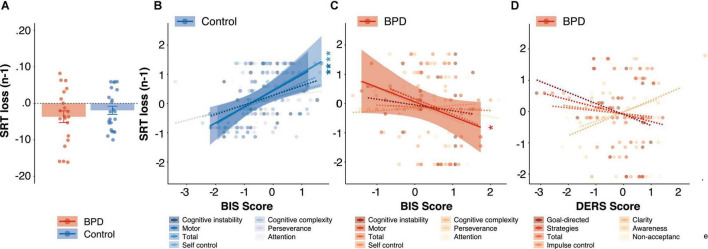
Reaction times following loss (unrewarded) trials. **(A)** Group differences in reaction times following loss (unrewarded) trials. The relationship between SRT (loss) and BIS impulsivity scores in **(B)** control and **(C)** BPD participants. **(D)** The relationship between SRT (loss) and DERS emotion dysregulation scores in BPD participants. In panels **(B–D)**, *z*-scores are shown to allow for visualization across different subscales, standardized beta coefficients from linear models are reported, and solid and dashed lines indicate significant and non-significant regressions, respectively, and reported *p* values are Bonferroni corrected. * *p* < 0.05, ** *p* < 0.01.

Median SRTs did not differ between groups (β = 0.30, *t* = 1.06, *p* = 0.29, *p_*Bonferroni*_* = 1.00), but SRTs were modulated by trial outcome such that they were significantly longer following unrewarded trials as compared to rewarded trials in both the BPD group [Figure 4A; unrewarded (*n*-1) *M* = –367 ms, *SE* = 159 ms; rewarded (*n*-1) *M* = –194 ms, *SE* = 141 ms, β = 0.74, *t* = 7.14, *p* < 0.001] and the control group [Figure 4A; unrewarded (*n*-1) *M* = –191 ms, *SE* = 112 ms; rewarded (*n-*1) *M* = –146 ms, *SE* = 141 ms, β = 0.74, *t* = 7.14, *p* < 0.001], but SRT did not change as a function of group following either unrewarded trials (β = 0.14, *t* = 0.44, *p* = 0.66, *p_*Bonferroni*_* = 1.00) or rewarded trials (β = –0.13, *t* = –0.43, *p* = 0.67, *p_*Bonferroni*_* = 1.00).

**FIGURE 5:**
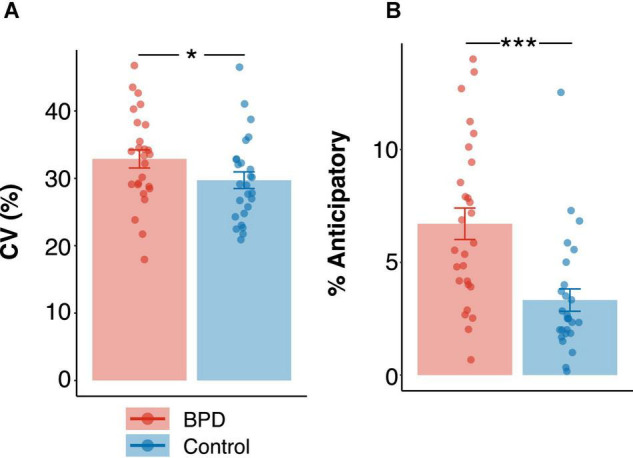
Group differences in **(A)** coefficient of variation in reaction times (%) and **(B)** percentage of anticipatory trials. Standardized beta coefficients from linear models are reported. *p* values in panel **(A)** are uncorrected and *p* values in panel **(B)** are Bonferroni corrected. * *p* < 0.05, *** *p* < 0.001.

Coefficient of variation was higher in the BPD group (Figure 5A and Table 3; *M* = 32.88, *SE* = 1.34) as compared to the control group (Figure 5A and Table 3; *M* = 29.72 ms, *SE* = 1.23 ms; β = –0.50, *t* = –1.99, *p* < 0.05, *p_*Bonferroni*_* = 0.20), though this failed to reach criteria for multiple comparisons. Additionally, the percentage of anticipatory trials was significantly higher in the BPD group (Figure 5B and Table 3; *M* = 6.71%, *SE* = 0.69%) as compared to the control group (Figure 5B and Table 3; *M* = 3.32%, *SE* = 0.49%; β = –0.92, *t* = –3.73, *p* < 0.001, *p_*Bonferroni*_* < 0.001).

No response time variables differed as a function of stimulant medication or ADHD diagnosis in BPD (all *p* > 0.05). We did not observe any significant effects of age (all *p* > 0.05), and age was modeled as a covariate in all group analyses. Finally, we did not observe any significant associations with overall BPD pathology as measured by the Borderline Symptoms List (BSL; Table 2, see Supplementary Section).

### Relationship Between Impulsivity and Matching Pennies Performance

Total BIS scores were higher in the BPD group (*M* = 80.81, *SE* = 1.88), compared to control participants (*M* = 64.55, *SE* = 2.15; β = –1.28, *t* = –5.87, *p* < 0.001). Broken down into the individual subscales, individuals with BPD scored significantly higher on all subscales (all *p* < 0.05, mean scores reported in Table 1). BIS scores did not differ as a function of age (all *p* > 0.05) with the exception of BIS self-control, which decreased with age (β = –2.14, *t* = –2.14, *p* = 0.04). However, there was no significant age by group interaction (β = 0.38, *t* = 1.48, *p* = 0.15), indicating that age effects were similar across both groups. Nonetheless, age was modeled as a covariate in all analyses. Several BIS scores were higher among BPD patients with ADHD (*n* = 14), including BIS Total (β = 0.77, *t* = 3.18, *p* = 0.004), BIS Motor (β = 0.77, *t* = 2.35, *p* = 0.03), and BIS Attention (β = 0.83, *t* = 3.47, *p* = 0.002) scores, however, we found no significant effect of stimulant medication on BIS scores (all *p* < 0.05). For all significant relationships among BPD patients, we additionally modeled ADHD diagnosis and stimulus medication to ascertain whether these relationships were being driven by patients with ADHD.

#### Probability of Reward

We found a significant interaction between group and BIS-MO on overall reward rate (β = –0.99, *t* = –3.49, *p* = 0.001, *p_*Bonferroni*_* = 0.005), and *post hoc* tests revealed a significant positive association between BIS-MO scores and reward rate in the BPD group (Figure 2C; β = 0.82, *t* = 4.03, *p* < 0.001), but a non-significant negative association between BIS-MO scores and reward rate in the control group (Figure 2B; β = –0.19, *t* = –0.90, *p* = 0.38). The main effect of BIS-MO on reward rate did not meet criteria for multiple comparisons (β = 0.34, *t* = 2.13, *p* = 0.04, *p_*Bonferroni*_* = 0.20).

We found a significant interaction between group and BIS-CI on reward rate (β = –1.07, *t* = –3.43, *p* = 0.001, *p_*Bonferroni*_* = 0.005), and *post hoc* tests revealed a significant positive association between BIS-CI scores and reward rate in the BPD group (Figure 2C; β = 0.77, *t* = 2.87, *p* = 0.01). On the other hand, control participants showed a trend toward a negative relationship between BIS-CI and reward rate (Figure 2B; β = –0.35, *t* = –1.93, *p* = 0.06). We did not observe a significant main effect of BIS-CI on reward rate (β = 0.10, *t* = 0.59, *p* = 0.56, *p_*Bonferroni*_* = 1.00).

#### Response Time Variables

We found a significant interaction between group and BIS-Total on SRTs following unrewarded trials (β = 1.21, *t* = 3.19, *p* = 0.002, *p_*Bonferroni*_* = 0.008), and *post hoc* tests revealed a trend-level negative association between BIS-Total and unrewarded SRTs in the BPD group (Figure 4C; β = –0.69, *t* = –1.78, *p* = 0.09) and a significant positive association in the control group (Figure 4B; β = 0.57, *t* = 3.31, *p* = 0.003). There was no significant main effect of BIS Total (β = 0.15, *t* = 0.73, *p* = 0.47, *p_*Bonferroni*_* = 1.00).

We found a significant interaction between group and BIS-MO scores on SRTs following unrewarded trials (β = 1.03, *t* = 3.47, *p* = 0.001, *p_*Bonferroni*_* = 0.004), and *post hoc* tests revealed a significant negative association between BIS-MO and unrewarded SRTs in the BPD group (Figure 4C; β = –0.56, *t* = –2.18, *p* = 0.04) and a significant positive association in the control group (Figure 4B; β = 0.53, *t* = 3.03, *p* = 0.01). There was no significant main effect of BIS-MO (β = –0.02, *t* = –0.12, *p* = 0.91, *p_*Bonferroni*_* = 1.00).

**FIGURE 6:**
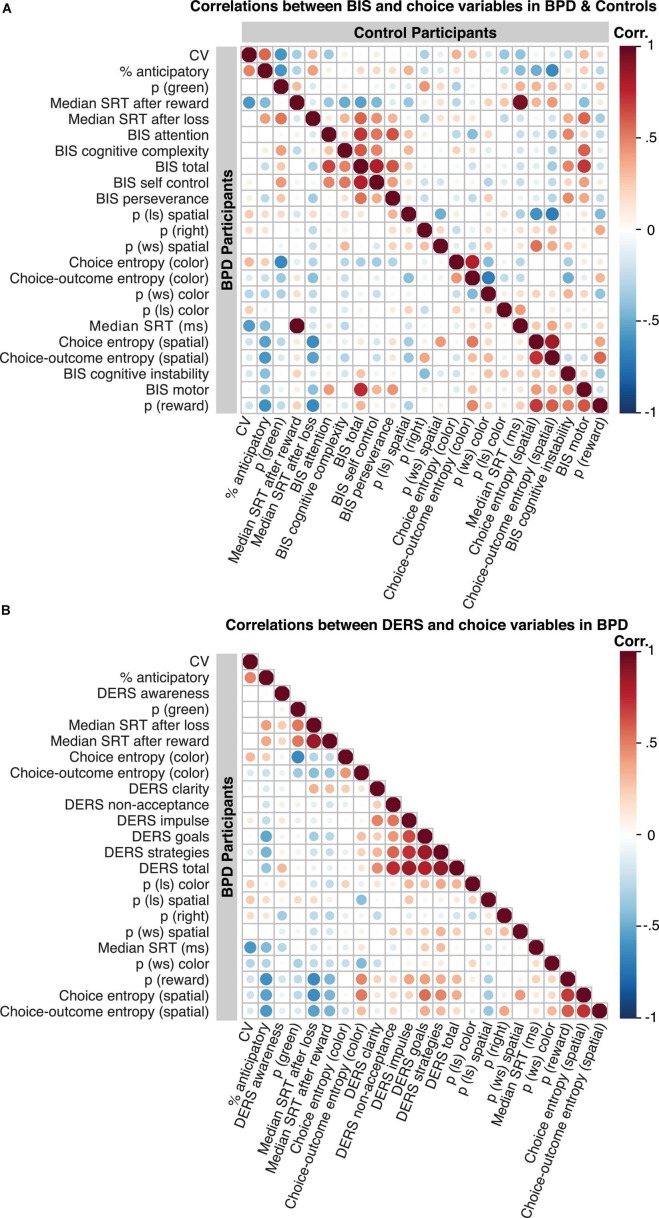
Correlation matrices showing relationships among behavioral variables and assessment data. **(A)** Relationship between BIS scores and choice behavior in BPD is shown in the bottom panel and in control participants in the top panel. **(B)** Relationship between DERS scores and choice behavior in BPD (DERS was not conducted in control participants). “Hot” and “cold” colors reflect positive and negative relationships among variables, respectively. The intensity of the color reflects the correlation value, and the size of the circles reflect the *p* values. *P* values are uncorrected.

We observed no significant associations between task performance variables and the BIS attention, self-control, cognitive complexity, and perseverance subscales (all *p* < 0.05, Figure 6A), and all relationships remained following the inclusion of stimulus medication and ADHD diagnosis (all *p* < 0.05) in the BPD group.

### Relationship Between Difficulties in Emotional Regulation and Matching Pennies Performance in Borderline Personality Disorder

Difficulties in emotional regulation assessments were conducted in the BPD group only, and we observed no significant effects of age, ADHD, or stimulant medication in DERS scores across any subscale (all *p* > 0.05).

#### Reward Rate

Difficulties in emotional regulation goals (Figure 2D; β = 0.47, *t* = 2.20, *p* = 0.04, *p_*Bonferroni*_* = 0.20) and DERS impulse (Figure 2D; β = 0.45, *t* = 2.11, *p* = 0.05, *p_*Bonferroni*_* = 0.25) were positively associated with reward rate, although neither survived multiple comparisons correction.

#### Entropy (Spatial)

Difficulties in emotional regulation goals (Figure 6B; β = 0.62, *t* = 3.52, *p* = 0.002, *p_*Bonferroni*_* = 0.01) and DERS strategies (Figure 6B; β = 0.50, *t* = 2.50, *p* = 0.02, *p_*Bonferroni*_* = 0.10) were positively associated with spatial choice entropy, although the latter failed to survive multiple comparisons.

#### Response Time Variables

We also found a significant association of DERS Goals with the percentage of anticipatory trials (Figure 6B; β = –0.53, *t* = –2.94, *p* = 0.01, *p_*Bonferroni*_* = 0.04).

We observed no significant associations between task performance variables and the DERS clarity, non-acceptance, and awareness subscales (all *p* < 0.05, Figure 6B), and all relationships remained following the inclusion of stimulus medication and ADHD diagnosis (all *p* < 0.05).

#### Correspondence Between Difficulties in Emotional Regulation Scale and Borderline Personality Disorder

To understand the common mechanisms by which our assessment data affects choice patterns during matching pennies, we highlight critical associations here. Importantly, BIS-CI was significantly associated with the majority of DERS subscales (all *p* < 0.05, with the exception of non-accept and awareness), and we found no other associations between DERS and BIS scores across any other subscale.

### Exploratory Analyses: Relationship Between Strategic Assessment and Matching Pennies Performance

#### Attention to Outcome

As a group, individuals with BPD reported paying significantly less attention to outcome during the mixed-strategy game (Supplementary Figure 1A; *M* = 3.55, *SE* = 0.23) as compared to control participants (Supplementary Figure 1A; *M* = 4.30, *SE* = 0.13; β = 0.77, *t* = 2.95, *p* = 0.005). Otherwise, BPD and control participants did not differ in self-report strategies (Table 1), and self-report strategies did not differ as a function of age (in either group) or ADHD diagnosis and stimulant medication in BPD participants (all *p* > 0.05).

##### Reward Rate

We saw a trend-level main effect of “attention to outcome” on reward rate (β = 0.35, *t* = 2.44, *p* = 0.02, *p*_*Bonferroni*_ = 0.10), with more attention to outcome associated with marginally increased reward rate. We did not observe a significant attention to outcome by group interaction on reward rate (β = –0.06, *t* = –0.17, *p* = 0.87, *p*_*Bonferroni*_ = 1.00).

##### Spatial Choice Entropy

We also saw a significant main effect of “attention to outcome” on choice entropy (spatial; Supplementary Figure 2B; β = 0.45, *t* = 3.25, *p* = 0.002, *p*_*Bonferroni*_ = 0.01), with higher attention to outcome being associated with increased entropy (i.e., decreased predictabilities). We did not observe a significant attention to outcome by group interaction on entropy (β = –0.06, *t* = –0.20, *p* = 0.84, *p*_*Bonferroni*_ = 1.00).

##### Anticipatory Trials

We saw a significant main effect of “attention to outcome” on anticipatory trials (β = –0.36, *t* = –2.86, *p* = 0.01, *p*_*Bonferroni*_ = 0.05), with more attention to outcome being associated with fewer anticipatory trials. We did not observe a significant attention to outcome by group interaction on anticipatory trials (β = –0.04, *t* = –0.16, *p* = 0.87, *p*_*Bonferroni*_ = 1.00).

#### Human vs. Computer

We observed a significant main effect of human vs. computer on p (WSLS color; β = 0.37, *t* = 2.93, *p* = 0.01, *p*_*Bonferroni*_ = 0.05), with those reporting that they would play differently against a human opponent showing increases in p(WSLS). There was no significant group by assessment interaction (β = 0.002, *t* = 0.01, *p* = 0.99, *p*_*Bonferroni*_ = 1.00). Follow-up tests looking at win-stay and lose-shift separately revealed that this was being driven by an association with probability of lose-shift [p(LS)], as we saw a significant main effect of this item and lose-shift (Supplementary Figure 2B; β = 9.39, *t* = 3.15, *p* = 0.002, *p*_*Bonferroni*_ = 0.01), with those reporting that they would play differently against a human opponent showing increases in p(LS). We saw no significant group by assessment interaction (β = 0.08, *t* = 0.31, *p* = 0.76, *p*_*Bonferroni*_ = 1.00). We did not see a significant association with win-stay strategies (β = 0.15, *t* = 1.13, *p* = 0.26, *p*_*Bonferroni*_ = 1.00), or a significant interaction with group (β = –0.06, *t* = –0.21, *p* = 0.84, *p*_*Bonferroni*_ = 1.00).

We did not observe any significant effects of age (all *p* > 0.05), and age was modeled as a covariate in all analyses.

#### Correspondence Between Strategic Assessment, Difficulties in Emotional Regulation Scale, and Impulsivity in Borderline Personality Disorder

Intriguingly, we found strong positive associations between attention to outcome and the majority of DERS subscales in the BPD group (Supplementary Figure 1C), including DERS total (β = 0.63, *t* = 3.20, *p* = 0.004), DERS goals (β = 0.60, *t* = 3.02, *p* = 0.01), DERS strategies (β = 0.56, *t* = 2.69, *p* = 0.01), DERS impulse (β = 0.51, *t* = 2.41, *p* = 0.02), DERS non-acceptance (β = 0.55, *t* = 2.62, *p* = 0.02), with the exception of DERS awareness (β = 0.001, *t* = 0.001, *p* = 1.00) and DERS clarity (β = 0.42, *t* = 1.91, *p* = 0.07). We observed no significant associations between the strategic assessment and the BIS across any subscale (all *p* < 0.05).

## Discussion

In this article, we examined mixed-strategy decision-making in female adolescent outpatients diagnosed with Borderline Personality Disorder (BPD). In particular, we investigated whether adolescents with BPD differed from age and sex matched control participants in their ability to engage in a strategic competition that encouraged randomization in choice patterns (i.e., suppressing choice biases), and whether two core features of BPD, impulsivity and emotional dysregulation, underscored individual differences in choice behavior. We found that the BPD patients as a group showed fewer win-stay, lose-shift (WSLS) biases (Figure 3), particularly lose-shift (LS) biases, increased variability in reaction times (CV; Figure 5A) and more anticipatory decisions (Figure 5B), despite having comparable reward rates (Figure 2A) relative to control participants. Critically, we found that a subset of BPD participants with high levels of impulsivity showed higher overall reward rates (Figure 2C) and greater modulation of reaction times by outcome (Figure 4C), particularly following loss trials, relative to control (Figures 2B, 4B) and BPD participants with lower levels of impulsivity. Additionally, BPD participants with higher levels of emotional dysregulation showed marginally increased reward rates (Figure 2D) and increased entropy in choice patterns (Figure 6B), which might be driven by increasing vigilance to outcome information during the task (Supplementary Figure 1). These results may suggest that adolescents with BPD show changes in choice behavior during Matching Pennies, and that impulsivity and emotional dysregulation potentially contribute to variability in mixed-strategy decision-making in BPD.

### Group Differences in Matching Pennies Performance

The finding that adolescents with BPD had a greater propensity for variability in reaction times (Figure 5A) and a greater number of anticipatory trials (Figure 5B) is in line with prior studies showing increased anticipatory decisions during a stop-signal delay task (Nigg et al., 2005; Coffey et al., 2011), and during the pro- and anti-saccade task (Calancie et al., *in preparation*) in BPD, and could be related to behavioral dis-inhibition (Nigg et al., 2005), although overall response times did not differ between groups (Table 3). Likewise, increased variability (also referred to as “increased noise” in the literature) is a common observance across several psychological illnesses and personality psychopathologies (Willcutt et al., 2008; Peters and Büchel, 2011; Kunisato et al., 2012; Robinson and Chase, 2017), including BPD (Kaiser et al., 2008). The increased variation in response time variables in the BPD group could therefore reflect increased variability with emerging psychopathology, and also suggests that our task may be sensitive to known behavioral changes in BPD.

We also found *reduced* WSLS bias in BPD relative to controls, which was being driven by a decrease in LS bias (Figure 3A), which may have provided a benefit to Matching Pennies performance as the strategic nature of our task incentivizes entropy in choice behavior (i.e., fewer reinforcement learning biases) in order to evade exploitation by one’s opponent (Rapoport and Budescu, 1992; Azar and Bar-Eli, 2011; Gauriot et al., 2016; Thaler, 2016). Despite this decrease in LS strategies, overall performance (as indicated by reward rates) was comparable among groups (Figure 2A), which is likely due to the wide array of choice biases and factors that could contribute to overall task performance (i.e., that could be exploited by the computerized opponent), the aggregate of which is reflected in overall reward rates. For example, although BPD participants showed decreased LS, they also showed increased CV and anticipatory decisions that appeared to considerably serve as a detriment to performance (Figure 6A). Further, the BPD group reported paying less attention to outcome information during the game (Supplementary Figure 1A), which was also associated with decreased reward rate. Thus, decreased LS may have partially counteracted the detrimental effects of increased CV, anticipatory choices, and reduced attention to outcome in BPD, resulting in comparable reward rates across groups.

In contrast to traditional decision-making paradigms, decreased LS biases served as adaptive during Matching Pennies. However, this reduced LS could be reflective, in part, of changes in limbic processes related to feedback processing (Goyer et al., 1994; Lyoo et al., 1998; Tebartz Van Elst et al., 2003; Silbersweig et al., 2007; Schuermann et al., 2011; Svaldi et al., 2012; Schulze et al., 2016; Paret et al., 2017), particularly with regard to outcome evaluation when a potential loss is present (de Bruijn et al., 2006; Vega et al., 2013; Sánchez-Navarro et al., 2014) and the ability to adjust behavior accordingly (de Bruijn et al., 2006; Vega et al., 2013). The reduced LS bias in BPD observed in the current study could potentially indicate aberrant PE following negative outcomes, which may contribute to disadvantageous decision-making tendencies observed in BPD in other decision-making paradigms (de Bruijn et al., 2006; Haaland and Landro, 2007; Schuermann et al., 2011; Vega et al., 2013), although future work is needed to support this hypothesis. Likewise, the reduced bias could be linked to delay discounting findings in BPD (Volker et al., 2008; Lawrence et al., 2010; Coffey et al., 2011; Barker et al., 2015; Krause-Utz et al., 2016; Maraz et al., 2016), as faster discounting of reinforcers may manifest as fewer choice biases. Alternatively, the possibility exists that reduced LS could be related to a more deliberative strategy (i.e., suppression of choice biases in an attempt to be random) in the BPD participants. Interestingly, our exploratory analyses revealed a positive association between LS and the “human vs. computer” item on the strategic questionnaire that assessed whether participants would approach the game differently if playing against a human opponent (Supplementary Figure 2B). Overall, while BPD patients and controls alike reported that they would indeed play differently against a human (Table 1), those who reported that they would approach the game similarly showed *decreased* LS biases. This finding, while exploratory and based on a post-game questionnaire, may provide evidence that individuals with BPD (who showed decreased LS biases as a group) potentially engaged different motivational processes that facilitated the suppression of choice biases and/or randomization over choice options in order to gain strategic advantage, in line with studies showing that knowledge of the task may contribute to variance in behavioral performance in BPD (Paret et al., 2017).

### Individual Differences in Impulsivity and Matching Pennies Performance

Impulsivity is a tendency to react to external stimuli, often quickly, without fully considering its consequences (Kim and Lee, 2011). We observed differential effects of impulsivity measures in Matching Pennies performance across groups; in the BPD group, higher levels of impulsivity, particularly cognitive instability and motor impulsivity, were associated with *increased* reward rate (Figure 2C), and greater modulation of SRT by outcome (Figure 4C), particularly unrewarded (loss) outcomes, in that higher levels of impulsivity were related to *longer* SRTs following loss trials. On the other hand, these measures were associated with little change among reward rates in control participants (Figure 2B), and we observed the opposite relationship with regard to SRTs following loss outcomes in control participants in that higher levels of impulsivity were instead related to *shorter* SRTs following loss trials (Figure 4B). Of note, *longer* SRTs following loss trials was correlated with *increased* reward rate in both groups (Figure 6A), and *increased* entropy in the BPD group (Figure 6A), suggesting that slowing down following a loss outcome may have served as a benefit to overall task performance. In particular, two subscales contributed to these findings: (1) BIS-Cognitive instability, which reflects instability in thought processes (see section “Results” for more detail; Patton et al., 1995; Stanford et al., 2009); and (2) BIS-Motor, which, despite being classified as impulsive responding in the motor domain, it is worth noting that it is also comprised of several questions relating to impulsive spending behaviors (see section “Materials and Methods” for more detail; Patton et al., 1995; Stanford et al., 2009). Increased impulsivity may diminish the ability to track changing action-outcome contingencies (Kim and Lee, 2011), as has been seen in tasks assessing reinforcement/reversal learning in BPD (Paret et al., 2016, 2017). While this tendency may lead to dysfunction in real-world settings in BPD (Paret et al., 2017), during mixed-strategy games and other related strategic decision-making paradigms, increased behavioral variability (i.e., randomness) can be adaptive (Tervo et al., 2014), and increased impulsivity may therefore have had a facilitatory effect on the ability to produce unpredictable choice sequences. Though difficult to distinguish between “noise” and randomness and strategic randomness, the observation that impulsivity was not related to either median SRTs or SRTs following rewarded trials but appeared to have a specific effect on SRTs following negative outcomes may suggest differential modulation of behavior in BPD participants with higher levels of impulsivity (rather than simply increased noise). In healthy individuals, increased impulsivity was associated with speeding up after a negative outcome, however, in BPD, the opposite pattern was observed whereby they slowed down after a negative outcome. BPD participants have been shown to have slower reaction times following negative stimuli relative to neutral [during an emotional stroop task; Arntz et al., 2000; Sieswerda et al., 2007a,b; Carpenter and Trull, 2012; Winter et al., 2015; Krause-Utz et al., 2018), which might be related to hypersensitivity to negative stimuli (Bertsch et al., 2018). Furthermore, impulsivity has been associated with post-error slowing and increased error related negativity (Hill et al., 2016). Our observation that higher levels of impulsivity in BPD were related to slowing down following unrewarded outcomes may be related to an increased expectancy of receiving punishments, and an adaptive/compensatory response to avoid them (i.e., to slow down; Hill et al., 2016), though future work is needed to assess this possibility.

Although the divergent associations between BIS scores and choice behavior across groups observed in the current study was unexpected, a few potential (non-mutually exclusive) explanations exist for these discrepancies. First, BIS scores were significantly lower in control participants compared to BPD, and therefore, the lack of associations in the control group could indicate of a floor effect and may suggest that impulsivity levels are too low in this cohort to detect associations with decision-making behavior that might emerge with higher levels of impulsivity. On the other hand, it is possible that BPD participants with high levels of impulsivity may have developed compensatory decision-making strategies that were reflected during our task (i.e., reflected in the modulation of SRTs by negative outcomes; Paret et al., 2017). In addition, studies have shown the impulsivity may modulate ventral striatal responses to rewards differently in healthy individuals as compared to psychiatric populations, including BPD (Herbort et al., 2016). Whereas in healthy individuals, increased impulsivity was related to increased ventral striatal responses (Hariri et al., 2006; Forbes et al., 2009; Plichta and Scheres, 2014; Herbort et al., 2016), in patient groups such as substance use, ADHD, and BPD, impulsivity was related to decreased ventral striatal responses (Beck et al., 2009; Plichta and Scheres, 2014; Herbort et al., 2016). Therefore, it is possible that differences in reward-related processing may partially underlie the opposing effects of impulsivity on choice behavior observed in the current study. Second, different relationships in controls and BPD may suggest that other pathological processes (overall psychiatric liability, for example) that are associated with BPD are contributing to the observed effect of impulsivity on choice behavior. In support of this notion, we found similar associations with high levels of emotional dysregulation in BPD (Figures 2D, 4D, see below for discussion), particularly across the goals, impulsivity, and strategies subscales, and DERS scores were highly correlated with BIS scores in BPD, suggesting that these two scales may be tapping into similar constructs underlying BPD pathology. In line with consistent findings in BPD showing exacerbated impulsivity in the face of negative affect and emotional regulation difficulties (Koenigsberg et al., 2001; Chapman et al., 2008; Peters et al., 2013; Sebastian et al., 2013; Scott et al., 2014), our findings may suggest that impulsivity exerts it’s influence on strategic choice behavior in part through the mediating role of emotion dysregulation, although we did not assess this possibility directly.

### Individual Differences in Emotion Dysregulation and Matching Pennies Performance

Emotion dysregulation may disrupt self-regulation and deliberative processes in BPD (Sharp et al., 2011; Hallquist et al., 2018), including mentalization (Sharp et al., 2011; Henco et al., 2020). We found that reward rates were marginally increased as a function of higher levels of emotion dysregulation in the BPD group, particularly, across the goals subscale, which reflects a lack of ability to engage in goal-directed behaviors when emotionally distressed (see section “Materials and Methods” for more detail; Gratz and Roemer, 2004; Herr et al., 2013), and the impulse control subscale, which reflects difficulties controlling impulsive behaviors when emotionally distressed (see section “Materials and Methods” for more detail; Gratz and Roemer, 2004; Herr et al., 2013). The latter is in confirmation of the BIS findings in BPD. Additionally, and potentially contributing to the relationship between DERS and reward rate, we found that choice entropy (in the spatial domain) also increased with higher levels of emotion dysregulation across the goals (Figure 6B) and strategies (Figure 6B) subscales. Although it is challenging to ascertain whether individuals with higher levels of emotional dysregulation deliberately employed the use of strategic tactics to reduce choice biases (increase entropy) or whether this reflects non-strategic randomness (“noise”), we found that higher levels of emotional dysregulation were associated with increased attention to outcome information (based on our exploratory analyses, Supplementary Figure 1C), which was itself associated with marginally increased reward rates and choice entropy. Although speculative, these results may suggest that increased levels of emotional dysregulation may contribute to strategic choice behavior in part by enhancing vigilance to outcome information.

## Conclusion and Next Steps

Economic exchange games are gaining significant traction in understanding deficits in interpersonal functioning across a number of psychiatric conditions (Euler et al., 2019). Game theoretical studies in BPD have found that behaviors during social interactions appear to be *less* modulated by social signals, which in particular contexts, may appear as greater “rationality” in choice (Jeung et al., 2016). While the interpersonal deficits associated with BPD create conflict across a number of domains, in some cases, may have a faciliatory effect on behaving in a self-interested manner in social and economic interactions (Jeung et al., 2016). While behavior during cooperative contexts has been characterized in BPD (King-casas et al., 2008; Sharp, 2012), to our knowledge, this is the first study to examine behavior during a competitive context in BPD. We found that increased emotional dysregulation and increased impulsivity in BPD may facilitate decreased choice biases in this competitive context, although future studies are required to further ascertain whether this is a deliberate process/strategy or a reflection of increased psychopathology more generally (Mukherjee and Kable, 2014).

In summary, our results provide a novel account of how two main constructs underlying BPD pathology, impulsivity and emotional dysregulation, affect decision-making in a competitive context. Research supports that individuals with higher clinical indices of impulsivity and affective dysregulation, compared to the BPD clinical group average, experience high therapy-burnout (Yeomans et al., 1994), are more likely to utilize high-cost centers (i.e., ER, inpatient hospitalization) to manage symptoms, and are more likely to attempt suicide (Yeomans et al., 1994; Black et al., 2004; Lieb et al., 2004; Speranza et al., 2011). Despite this, these individuals access the same therapy regimen as other BPD patients (DBT and interpersonal therapy). If replicated in a larger sample, it is possible that the differences in mixed-strategy choice behavior observed in individuals with high levels of impulsivity and emotional dysregulation could be leveraged to identify high-risk patients and streamline individuals into targeted treatment regimens.

## Limitations

Several limitations in the present study should be noted. First, our BPD sample was comprised of all treatment-seeking female adolescents, which may limit generalizability to broader BPD populations. Given that patients were recruited from Dialectical Behavioral Therapy (DBT) groups, it is possible that treatment effects may have contributed to some of the observed results. Several aspects of strategic choice behavior were not significantly different across groups, including overall reward rate, which could suggest that BPD participants, particularly the high impulsivity and emotional dysregulation groups, may have been able to overcome deficits through the use of compensatory strategies (Paret et al., 2017), for example, by increasing vigilance to outcome information, or by opting for the use of random strategies. Another possibility is that while the current study captured the dynamic and interactive nature of complex, real-world strategic engagements, given the use of a computerized opponent (that participants were made aware of), our task only partially captured the *social* components (Rilling et al., 2002; Spiliopoulos, 2008; Vickery et al., 2015; Parr et al., 2020), thus may not be sensitive to the full range of interpersonal dysfunction typically associated with BPD. Future studies should therefore seek to understand whether strategic game-play against a human opponent stimulates different mentalization processes and is more sensitive to maladaptive decision-making tendencies in adolescents with BPD. Another factor limiting generalizability is that our paradigm did not include a reinforcement learning task for comparison that would allow us to discern whether an increase in unpredictable choice patterns during Matching Pennies was related to explicit suppression of reinforcement learning biases (i.e., strategic randomness) or whether it was related to an inability to assess and choose among options varying in value that would likely result in poorer task performance in other contexts (i.e., during the Iowa Gambling Task; Haaland and Landro, 2007; Schuermann et al., 2011). Future studies should seek to clarify whether seemingly improved performance with higher levels of impulsivity and emotional dysregulation in BPD are reflective of intentional (strategic) randomness or unintentional randomness that may be a reflection of psychopathology (Mukherjee and Kable, 2014). Another limitation in the current study is the lack of a secondary assessment of cognition or non-verbal intelligence that would allow us to disentangle changes in strategic choice behavior from general cognitive deficits in other contexts. Finally, given that this exploratory study was cross-sectional with a relatively small sample size and several of the effects observed in the current study were modest, we may have been underpowered to detect meaningful effects and therefore, an important area for future work is to extend this line of inquiry into a large, well-powered, sample using a longitudinal design (potentially in high-risk developmental samples) that explores the relationship between emergent BPD traits in adolescents and functional outcomes through to adulthood.

## Data Availability Statement

The raw data supporting the conclusions of this article will be made available by the authors, without undue reservation.

## Ethics Statement

The studies involving human participants were reviewed and approved by the Queen’s University Human Research Ethics Board, Kingston, ON, Canada. Written informed consent to participate in this study was provided by adults aged ≧ 18. Participants ages <18 years provided verbal assent, and written informed consent to participate in the study was provided by the participants’ legal guardian/next of kin.

## Author Contributions

AP, BC, and DM designed the experimental protocol. AP and OC performed research and collected data. AP, OC, and SK-K recruited and screened participants. AP, OC, and BC analyzed data. AP, OC, DM, and SK-K wrote the manuscript. All authors contributed to the article and approved the submitted version.

## Conflict of Interest

The authors declare that the research was conducted in the absence of any commercial or financial relationships that could be construed as a potential conflict of interest.

## Publisher’s Note

All claims expressed in this article are solely those of the authors and do not necessarily represent those of their affiliated organizations, or those of the publisher, the editors and the reviewers. Any product that may be evaluated in this article, or claim that may be made by its manufacturer, is not guaranteed or endorsed by the publisher.
