# Supplementary material for: Impulsivity and Emotional Dysregulation Predict Choice Behavior During a Mixed-Strategy Game in Adolescents With Borderline Personality Disorder
Source: Front Neurosci. 2022 Feb 14;15:667399. doi: 10.3389/fnins.2021.667399 (PMC8882924; doi:10.3389/fnins.2021.667399)
Supplement: Supplementary file 1 [file Image_1.pdf]

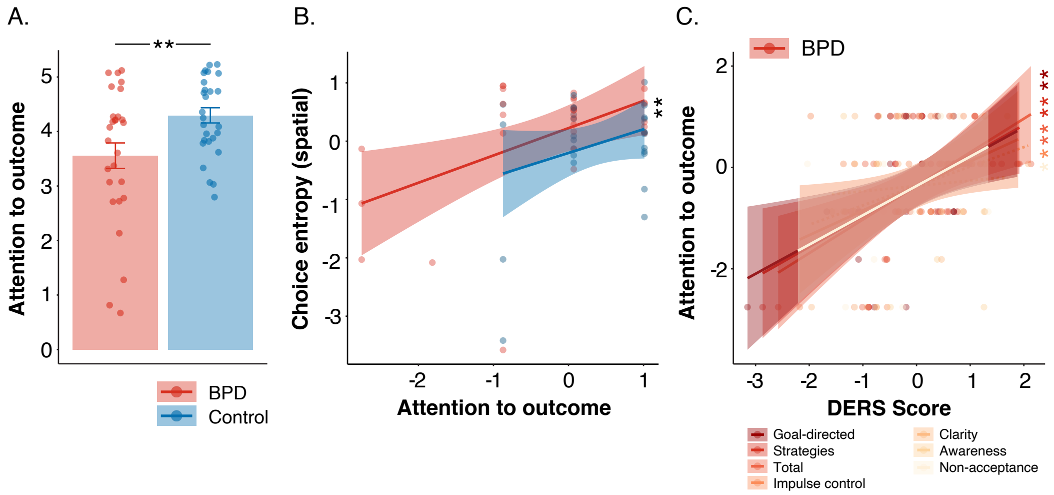

Figure S1. Attention to outcome and associations with DERS scores in BPD

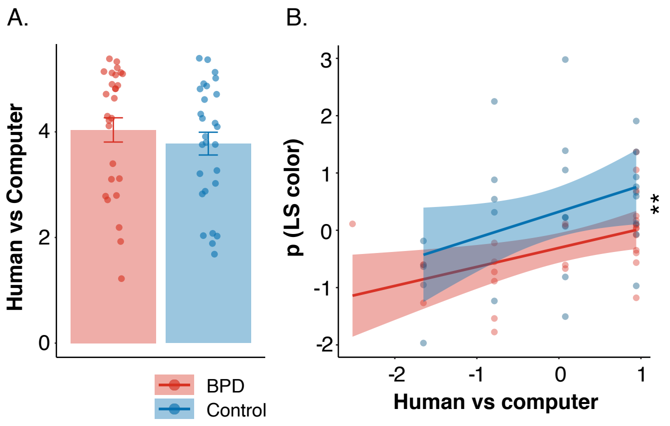

Figure S2. p (LS color) and associations with human vs computer item on strategic questionnaire
