# Supplementary material for: Impulsivity and Emotional Dysregulation Predict Choice Behavior During a Mixed-Strategy Game in Adolescents With Borderline Personality Disorder
Source: Front Neurosci. 2022 Feb 14;15:667399. doi: 10.3389/fnins.2021.667399 (PMC8882924; doi:10.3389/fnins.2021.667399)
Supplement: Supplementary file 2 [file Data_Sheet_1.docx]

**Relationship between Borderline Symptom List and Matching Pennies Performance.**

BSL did not change with age in the BPD participants (*M* = 53.52, *SE* = 4.88; $\beta$ = .38, *t* = 1.80, *p* = .08), and did not change as a function of stimulant medication ($\beta$ = -.76, *t* = -1.36, *p* = .19) or ADHD ($\beta$ = -.58, *t* = -1.55, *p* = .14). Reward rate did not differ as a function of BSL scores ($\beta$ = .14, *t* = .55, *p* = .59). Color choice entropy ($\beta$ = -.03, *t* = -.18, *p* = .86) and color choice-outcome entropy ($\beta$ = -.17, *t* = -1.02, *p* = .32) did not differ as a function of BSL scores. Spatial choice entropy ($\beta$ = .15, *t* = .63, *p* = .53) and spatial choice-outcome entropy ($\beta$ = .24, *t* = .91, *p* = .37) did not differ as a function of BSL scores. Color lose-shift (LS) strategies did not differ as a function of BSL scores ($\beta$ = .24, *t* = 1.70, *p* = .10) nor did color win-stay (WS) strategies ($\beta$ = .24, *t* = 1.56, *p* = .10). Spatial lose-shift (LS) strategies did not differ as a function of BSL scores ($\beta$ = .11, *t* = .44, *p* = .66) nor did win-stay (WS) strategies ($\beta$ = -.01, *t* = -.03, *p* = .97). Anticipatory rate ($\beta$ = -.27, *t* = -1.19, *p* = .25) and CV ($\beta$ = -.27, *t* = -1.12, *p* = .28) did not differ as a function of BSL scores. Median RTs (overall) did not differ as a function of BSL scores ($\beta$ = .06, *t* = .32, *p* = .75), nor did median RTs following rewarded trials ($\beta$ = .13, *t* = .54, *p* = .60), or median RTs following unrewarded trials ($\beta$ = -.03, *t* = -.14, *p* = .89). Finally, CV did not differ as a function of BSL scores ($\beta$ = .30, *t* = 1.17, *p* = .26).

**Supplementary Figure Captions**

**Figure S1.** Attention to Outcome During Matching Pennies. **(A)** Group differences in self-reported attention to outcome. **(B)** The relationship between attention to outcome and spatial choice entropy. **(C)** The relationship between attention to outcome and Difficulties in Emotion Regulation (DERS) scores in BPD participants. In B and C, z-scores are shown. Beta coefficients from linear effects models are reported, and solid and dashed lines indicate significant and non-significant regressions, respectively. Reported *p* values are Bonferroni corrected. * *p* < .05, ** *p* <.01, *** *p* < .001.

**Figure S2.** Human vs Computer Strategies During Matching Pennies. **(A)** Group differences in self-reported item “I would play differently against a human than the computer opponent”. **(B)** The relationship between human vs computer and the probability of lose-shift (p(LS)). In B, z-scores are shown. Beta coefficients from linear effects models are reported, and solid and dashed lines indicate significant regressions. Reported *p* values are Bonferroni corrected. ** *p* <.01.
